# EYE-ECG: An RCT of the influence of student characteristics and expert eye-tracking videos with cued retrospective reporting on students’ ECG interpretation skills

**DOI:** 10.3205/zma001695

**Published:** 2024-09-16

**Authors:** Aline D. Scherff, Stefan Kääb, Martin R. Fischer, Markus Berndt

**Affiliations:** 1 LMU University Hospital, LMU Munich, Institute of Medical Education, Munich, Germany; 2 LMU University Hospital, LMU Munich, Department of Medicine I, Munich, Germany

**Keywords:** eye-tracking, learning, electrocardiography, students, medical, clinical reasoning, diagnosis

## Abstract

**Objectives:**

Teaching of ECG interpretation frequently relies on visual schemas. However, subsequent student ECG interpretation skills are often poor. Expertise research shows that expert looking patterns frequently deviate from the steps taught in schema learning. The present study made a cardiology expert’s gaze interpreting ECGs visible – through eye-tracking videos with cued retrospective reporting (CRR) – and investigated the potential as an additional expert-driven route to improve medical students’ ECG interpretation skills.

**Methods:**

*N*=91 Medical students participated in the RCT of an ECG e-learning session aimed at medical students’ ECG interpretation skills gain, either receiving the newly developed eye-tracking video with CRR audio commentary materials (*n*=47) or studying via four clinical cases only (*n*=44). Three outcome scores relating to different aspects of ECG interpretation skills were derived from pre-post MCQ ECG tests. The effect of the EYE-ECG training and additional characteristics (e.g., prior experience, interest) on student ECG interpretation skills were evaluated using t-tests and multivariate linear regression.

**Results:**

A small, non-significant advantage of the EYE-ECG training signifying a tendency for greater knowledge gain was observed, compared to training as usual. In multivariate regression models, the predictive value of clinical case 1 was an unexpected finding warranting further exploration.

**Conclusion:**

Additional gains after an only 9-minute intervention using videos of expert’s real-time gaze pattern in combination with hearing their thought processes during ECG interpretation is a promising finding. Furthermore, a number of specific performance characteristics enabling students to best benefit from ECG training were identified and possible modifications to the learning intervention suggested.

## Zusammenfassung

**Hintergrund:**

Die Lehre der EKG-Befundung stützt sich häufig auf visuelle Schemata. Die resultierende EKG-Befundungskompetenz durch die Studierenden ist jedoch oft mangelhaft. Die Expertiseforschung zeigt, dass die Blickmuster von Experten häufig von den Schritten abweichen, welche beim Schema-Lernen vermittelt werden. Die vorliegende Studie visualisierte den Blick eines Kardiologieexperten bei der EKG-Befundung – durch Eye-Tracking-Videos mit Cued Retrospective Reporting (CRR) – und untersuchte das Potenzial als zusätzliche expertenbasierte Möglichkeit zur Verbesserung der EKG-Befundungskompetenz von Medizinstudierenden.

**Methodik:**

*N*=91 Medizinstudierende nahmen an der RCT einer EKG-Lernstudie teil, die darauf abzielte, die EKG-Befundungskompetenz von Medizinstudierenden zu verbessern. Sie erhielten entweder das Standard-Training (*n*=44) mit vier Lernfällen oder zusätzlich das neu entwickelte Eye-Tracking-Video mit CRR-Audiokommentarmaterial (*n*=47). Drei Befundungsscores, die sich auf verschiedene Aspekte der EKG-Befundungskompetenz beziehen, wurden aus den prä-post Multiple-Choice EKG-Tests entwickelt. Die Effekte des EYE-EKG Trainings und zusätzlicher Studierendenmerkmale (z. B. Vorerfahrung, Interesse) auf die EKG-Befundungskompetenz der Studierenden wurden mit Hilfe von t-Tests und multivariater linearer Regression bewertet.

**Ergebnisse:**

Es zeigte sich ein kleiner, nicht signifikanter Lernvorteil des EYE-EKG-Trainings, entsprechend einer Tendenz zu einem größeren Wissenszuwachs im Vergleich zum Standard-Training. In den durchgeführten multivariaten Regressionsmodellen war die prädiktive Rolle von Fall 1 ein unerwartetes Ergebnis, welches weiter exploriert werden muss.

**Fazit:**

Der zusätzliche Lernfortschritt nach einer nur 9-minütigen Intervention – bei welcher Videos von Echtzeit-Blickmustern eines Experten in Kombination mit der auditiven Präsentation des Denkprozesses während der EKG-Befundung verwendet wurden – sind ein vielversprechendes Ergebnis. Darüber hinaus wurde eine Reihe von spezifischen Leistungsmerkmalen identifiziert, die es den Studierenden ermöglichen, am besten vom EKG-Training zu profitieren. Mögliche Modifikationen der Lernintervention werden vorgeschlagen.

## 1. Introduction

### 1.1. Learning and teaching of ECG interpretation skills

Electrocardiography (ECG) interpretation is a clinically highly relevant part of the medical education curriculum and is considered an Entrustable Professional Activity that medical students need to master prior to graduation [1], [2].

There currently exists widespread agreement on the importance of ECGs as a diagnostic test, and the impact of accurate interpretations of patient cases is further illustrated by the finding that patient cases in which an ECG is of relevance are particularly prone to error and can lead to incorrect treatment [3]. For these reasons, ECG interpretation forms part of core undergraduate medical training [4], and accuracy of ECG interpretation and the improvement thereof is already a widely studied topic, with a 2020 meta-analysis including 10056 participants across 78 studies [5]. Crucially, it concludes that performance results are frequently poor with a derived mean accuracy of 42% for medical students, which improved to 75% for cardiologists. Overall, ECG interpretation holds continued potential for improvement throughout one’s medical professional career and developing early approaches to more successful skill learning could greatly positively impact the medical field. 

Available training methods have generally been found to moderately improve ECG interpretation skills and improvement has also been linked to a stable dose-effect relationship [5]. Yet, to date, there is no universally recognised gold standard how ECG interpretation skills should be taught and knowledge thereof assessed [6]. A common approach to the current teaching of ECG interpretation relies on didactically designed schemas or checklists, which can be used in the interpretation process as an organisational framework to ensure a professional and complete reporting. Nevertheless, relying on experts to develop interpretation schemas comes at the cost of being resource intensive while using students’ own schemas has been shown as more burdensome on their cognitive load [7].

Moreover, expertise research shows that with increasing levels of expertise, physicians deviate more and more from standard schemas, and in doing so arrive at more accurate diagnoses more quickly [8], [9], [10]. Similarly, expert knowledge in other domains, such as expertise vs. novice play in chess, is well characterised. It is therefore both of great academic interest and of interest to medical students as ECG novices to be able to experience and learn from examples of expert ECG interpretation as fully as possible. Methods such as eye-tracking, which visualise expert knowledge for medical teaching of ECG interpretation skills – without having to rely on standard schema learning – could offer a direct path (eye-tracking presents actual viewing patterns) to examples of good ECG interpretation skills.

### 1.2. Expertise in medical visualisations from eye-tracking and verbal reports

Beyond schema learning, differences between experts and novices in their approach to medical visualisations, such as ECGs, have been demonstrated through a variety of measures. A review by Gegenfurtner et al. [11] concludes that two of the major approaches to tease out differences in strategies have been tracking eye movements, and collecting and using verbal reports. These have been considered as mutually complementary, since eye-tracking is a relatively recent technology to demonstrate and share visual search and perception, and verbal reports permit insight into clinical reasoning and assign meaning to what one sees [12]. 

Among the main themes that have been considered for their potential differences between experts and novices are time on task, eye movement characteristics of experts, differences in visual attention, visual search patterns, and the effect of teaching visual search strategies (review in [13]).

Overall, expert cardiologists’ gaze is characterised by narrowly focused attention on few but relevant spots when interpreting ECGs and verbal reports already generated hypotheses including relevant diagnostic information [11].

However, less progress has been made in converting specific (expert-like) search strategies to measurable student skill gains via deliberate teaching: For instance, a study by Kok et al. showed that their training in X-ray viewing led to a more systematic search but did not result in a higher diagnostic precision as compared to a student group not using the proposed strategy [14].

Therefore, it has been proposed that novices – lacking *a* *priori* hypotheses allowing them to benefit from the efficient expert schemas – may benefit instead from an approach that directs learners’ attention to specific areas more generally [13] but lets them draw their own inferences. While some of this may be achieved via shared attention during face to face communication, using an eye-tracker for eye movement modelling by visualising expert scan paths [15], [16] makes this strategy available for self-paced digital learning. While this is of current appeal as a strategy suitable for COVID-19 contingencies where an expert teacher is not available, eye-tracking (unlike more traditional verbal lectures using laser pointers as aids) also has the added benefit of ensuring all cues are unambiguously presented and visible to learners at all times. Importantly, it presents interpretative viewing patterns *as* *they truly are* rather than how the expert teacher believes they should be presented.

### 1.3. Cued retrospective reporting

A promising method to support student learning processes is the combination of eye movement measurement (eye-tracking) and cued retrospective reporting (CRR, supported verbal explanation of eye movements) [17]. CRR is a specific form of a retrospective think-aloud protocol based on eye movements. As opposed to concurrent thinking-aloud methods, it asks subjects to verbally report their thoughts after completion of a task, in this scenario using their own previously recorded eye-tracking patterns as memory cues. The advantage of cued retrospective reporting is that it does not interfere with task performance [12], i.e., by avoiding the risk of increased task demands, divided attention, or altering intuitive viewing patterns to suit verbal descriptions. It has been suggested that contributors are generally sufficiently accurate in their retrospective reporting, and this is particularly true for short tasks lasting 5-10 seconds [18], such as the time it takes an expert to interpret an ECG. Moreover, Helle (2017) also postulates that retrospective verbalisation appears to be the preferable option for eliciting more in-depth explications for very short tasks where there is simply not enough time to verbalise, and this has also been demonstrated experimentally, showing that compared to other methods, cued retrospective reporting is qualitatively equivalent and produces a greater quantity of comments [12], [19].

Briefly, this method has been used successfully in a variety of learning scenarios including problem-solving [17], information-seeking tasks [19], [20], [21], and the interpretation of diagrams [22], but is an innovation in the context of ECG learning. Studies thus suggest that eye-tracking and CRR can act as a suitable method to support learning [15], [17], and the limited literature available on medical imagery would suggest that CRR currently poses a novel approach to medical teaching.

### 1.4. Aim of the present study

In summary, ECG interpretation skills are an important part of medical students’ curriculum but often rely on teaching via schema, with currently poor performance results. An alternative, novel approach to teaching and learning ECG interpretation skills may be achieved via a combined eye-tracking and retrospective thinking-aloud protocol.

The present study investigated the effects of eye-tracking videos with cued retrospective reporting by a cardiology expert interpreting ECGs on medical students’ ECG interpretation skills. In addition to the aforementioned evaluation of this new training intervention, students’ ECG interpretation skills and the potential contribution of CRR-based learning were evaluated against the background of multiple other characteristics contributing to their ECG interpretation skills. These included a range of both short and long term factors such as overall prior knowledge, current motivation, and three conceptualisations of ECG learning outcomes (initial identification of ECG features, avoiding errors, and reaching a fully correct diagnosis).

Research question one investigated the usefulness of CRR videos for student ECG learning, hypothesising that the intervention group (INT) would show greater ECG interpretation skills post-training as compared to training as usual (TAU). Research question two asked which characteristics could best predict post-training ECG interpretation skills, hypothesising that these skills could firstly be meaningfully predicted and secondly that specific sets of relevant characteristics would emerge depending on the interpretation strategy in focus.

## 2. Materials and methods

### 2.1. Design

The study was designed as randomised controlled trial of a learning intervention aimed at advanced medical students in study year four to five, testing the effects of expert eye-tracking videos with cued retrospective reporting on students’ ECG interpretation skills in the *intervention group (INT)* vs. the *training as usual group (TAU)*. Ethics approval was granted by the institutional review board.

### 2.2. Materials

Material development concerned the initial recording of the EYE-ECG video (see videoclip for an extract: attachment 1 ), an expert eye-tracking of ECG interpretation and creation of gaze pattern videos with CRR audio commentary (for a full description, see attachment 2 , point A).

#### 2.2.1. Learning intervention

Combining all 15 video clips – obtained via eye-tracked expert ECG interpretation – with their CRR audio commentary resulted in a single (EYE-ECG) video of 9m 13s, simultaneously presenting both the visible expert gaze and CRR audio commentary. The addition of this video constituted the only difference for participants of the INT vs. TAU group in the intervention phase described hereafter.

**Figure 1:**
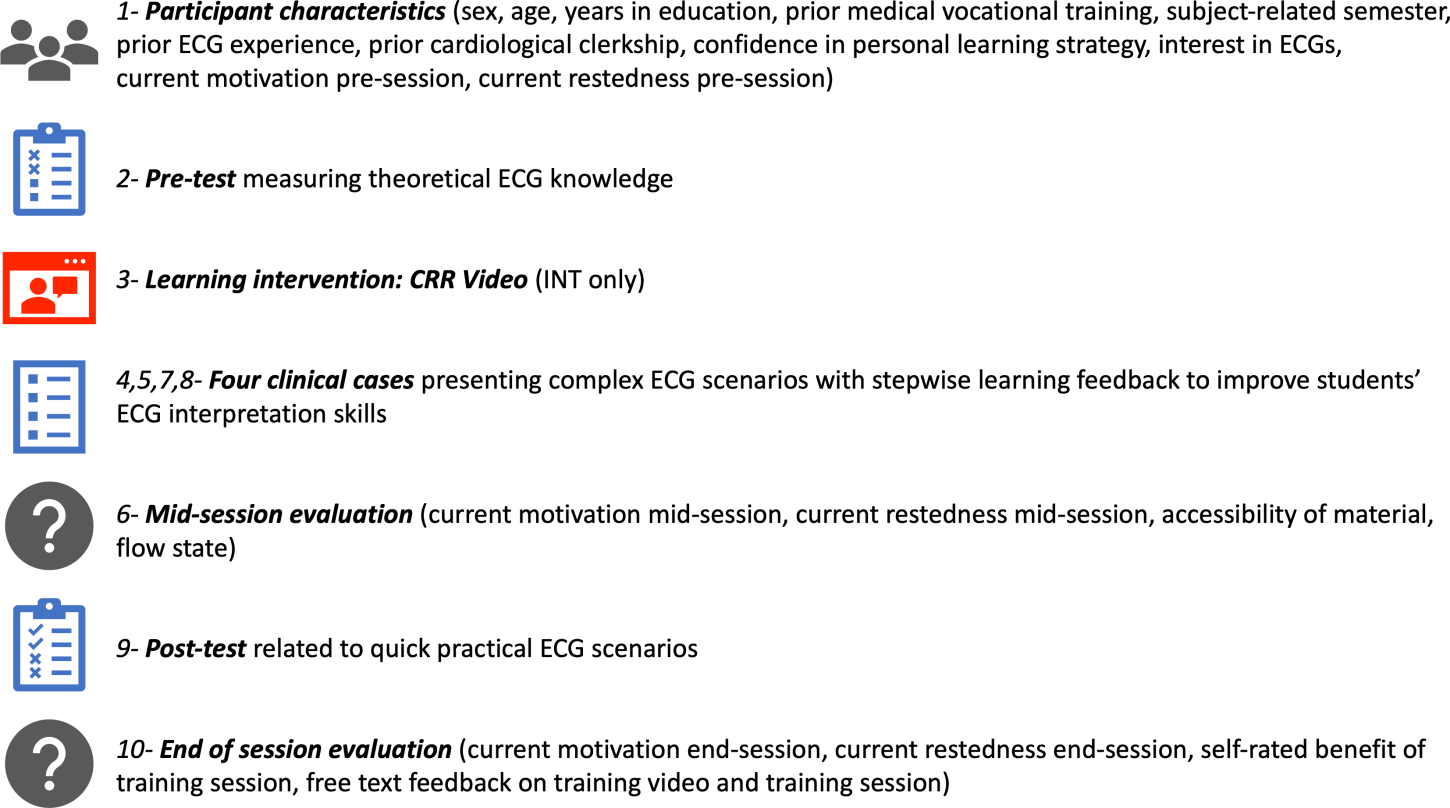
Overview of measures and procedure used in the study. Numbers refer to the order of presentation.

The intervention tested this newly developed video for its potential benefit regarding student ECG learning and was presented to the INT group after collection of participant characteristics and an ECG skills pre-test as the third component (see figure 1 (Fig. 1)).

#### 2.2.2. Measures

Student ECG training consisted of 9 (TAU) or 10 (INT) components respectively, scheduled as a single online session taking 5 hours at a maximum. All questionnaires testing ECG skills (i.e., see figure 1 (Fig. 1), components 2, 4, 5, 7, 8, 9) have been previously utilized and more detailed descriptions, including question content, answer options, as well as validation information, is available elsewhere [23], [24], [25]. Beyond the trialling of the EYE-ECG video as a learning tool, the second novel aspect of this study was the utilisation of the just-mentioned ECG skills components to investigate their usefulness within the framework of three different aspects of ECG interpretation skills (feature identification, answer choice strategies, clinical requirements). Thus, hereafter, for the readers’ convenience a concise summary of the self-rated scales is given, and a more thorough explication of participants’ ECG interpretation components is provided.

##### 2.2.2.1. Self-rated scales

At the beginning of the session (component 1), participants reported on their *interest in ECGs* and *confidence in their own learning strategy*. Interest contained 6 items with the item conferring the greatest item-total correlation relating to “I find the exploration of ECG interpretation challenges exciting” and a scale reliability of Cronbach’s *α*=.76. *Learning* (27 items, *α*=.90) queried, e.g., “I am good at identifying the times when I can learn best”. Mid-session data collection (component 6) included ratings of *accessibility of the material* (8 items, *α*=.81, “How easy or difficult did you find it to integrate the new information with what you already knew about the topic”) and *flow state* relating to the immersiveness of the experience (11 items, *α*=.83, “During the learning session so far, I found learning really exciting”). End of session evaluation (component 10) asked about participants’ perceived learning gain (*self-rated benefit from session*, 15 items, *α*=.85, “I would recommend practicing with this student ECG training to my fellow students”). Responses on all self-ratings were collected as 6-point Likert items and for easer interpretability, the sum totals derived were transformed into scores indicating 0-100% agreement. In addition, indicator items were collected asking about students’ extent of their *motivation* and *restedness *(0-100%) using a single item pre, mid, and post learning session (included in component 1, 6, 7, respectively).

##### 2.2.2.2. Student ECG interpretation skills

The *pre-test *(component 2) measuring theoretical ECG knowledge consisted of 42 items with 4 answer options each (e.g., “Which of the following PQ times is/are considered physiological? – 0,12s/ 0,11s/ 0,20s/ 0,21s”). The *4 clinical cases* (components 4, 5, 7, 8) each presented an image of an ECG that needed to be interpreted step by step both visually and clinically, guided by 4 test questions per case with a large, varying number of answer options and stepwise feedback. For example, for case 1, within a single question, participants were to select from a longlist all features that applied to the ECG shown regarding heart rate (3 options), rhythm (12), position of the heart (7), abnormalities of intervals (7), of amplitudes (3), and in the formation, propagation, and regression of electrical excitation of the heart muscle (5). The *post-test* containing quick practical ECG scenarios (component 9) consisted of 9 actual patient ECGs accompanied by a total of 69 question items with 4 answer options (e.g., “Given Ms. B.'s symptoms and ECG, what suspected diagnosis(/es) should be given preferential consideration? – Recent myocardial ischemia/pulmonary artery embolism/cardiomyopathy/electrolyte imbalance”).

##### 2.2.2.3. Calculation of interpretation skill scores

Central focus of the study (and its primary outcome) are the quantitative differences in ECG interpretation skill gain (0-100% pre vs. post scores) at the end of the training session. Of note, while all test contents regarding ECGs are fully compliant with the national medical curriculum and should therefore have been familiar subject matter, the purposeful design as multiple-choice questions with an unknown number of correct answers (instead of a single one) to be selected represented a novel challenge to participants that vastly increased potential combinations and thus test difficulty.

To assess various aspects of ECG skill gain, three outcome scores were derived, each signifying unique strategic aspects and learning features, allowing for different inferences to be drawn: A basic score (BS) summing up all correctly logged options as percentage correct was created to evaluate students’ ability to initially identify relevant ECG features from all potential combinations (example pre-test: 42 items *4 options=only 168 clickable fields overall but truly 2^4^=16 possible combinations per *each* of the 42 items; 67 answers factually correct; thus a participant logging e.g., 49 correct fields scored 49/67=0.73=73%). Second, a *relative score (RS)* representing the fraction of correctly against falsely selected options (e.g., 73% correct-25% false=48%), evaluating students’ certainty in their answers and correcting for lucky guessers vs. cagey responders. Third, a very difficult to achieve *conservative score (CS)* only awarding points for fully correct items (e.g., all correct options and no incorrect options selected per item; a maximum of 42 points for pre-test=100%) and CS pertained to the need for medical students upon reaching professional qualification to accurately identify and treat all patient symptoms in correct combination to guarantee best clinical practice and warrant patient safety. The three outcome conceptualisations were applied to all ECG skill components (i.e., see figure 1 (Fig. 1), components 2, 4, 5, 7, 8, 9): For example, modelling conducted on BS consistently included BS pre-test scores, BS clinical cases 1-4 scores and BS post-test scores; RS models were based on all RS scores, and CS models on CS scores throughout.

### 2.3. Procedure

All participants (*N*=91; INT *n*=47; TAU; *n*=44) were randomly allocated to their group and completed the online training session. This included collection of participant characteristics, an ECG pre-test, four clinical cases with an intercalated mid-session evaluation, ECG post-test and post-test evaluation (for components and order of presentation see figure 1 (Fig. 1)) in pandemic-respecting small batches in-person under standardized conditions at a distraction-free university PC cluster. All participants were current medical students at the LMU Munich, Germany. They were recruited via email, and partaking was an extra-curricular activity. All medical students met the requirement of successful completion of their university cardiology module but were not yet fully qualified medical doctors. The intervention group was instructed to fully watch the EYE-ECG video at least once and were free to watch it multiple times. All items of training components were in fixed order and answers could not be changed once submitted, as sometimes more information became available subsequently. Similar time on task and successful randomisation was shown (piloting and manipulation checks, see table 1 (Tab. 1) of results).

Statistical analysis utilised standard practice parametric testing, i.e., t-tests for groupwise comparisons and linear regression for multivariate modelling, as appropriate (for model building, see attachment 2 , point B statistical analyses and point C bivariate associations of model variables).

## 3. Results

### 3.1. Sample characteristics

Requiring all participants to have passed their cardiology exam prior to the study, the sample was typical of medical students towards the latter part of their studies and consisted of 76% females and 24% males aged *M*=24.14 (*SD*=3.04) years. They were approaching the end of their medical degree with a current semester of *M*=9.79 (*SD*=1.41) equating to 4^th^/5^th^ year of studies. A prior medical vocational training had been completed by 18%. Similarly, years in education (*M*=17.57, *SD*=2.27) reflected that they had tended to be in continuous formal education. A voluntary prior cardiological clerkship was reported by 35% of participants. An equally large proportion (29%) reported never having received any dedicated ECG training, while the remainder stated having received ECG training during their studies (31%) or through some combination of university, online, and external sources (40%). Participants estimated their mean quantity of previously independently interpreted ECGs as *M*=24.90, though this number differed greatly amongst them (*SD*=66.28), which was consistent with the data showing that participants included both complete novices without any prior independent practice and well-practiced students with an emergency medical technician (EMT) background. In addition, student feedback supported the notion of a varied sample, which was evident e.g. from participants stating as reasons for partaking a wide spectrum ranging from keen interest in a cardiology specialisation, to a strategic decision to utilise the training for revision, to counteracting a lack of ECG experience. Additional sample characteristics are presented in table 2 (Tab. 2).

### 3.2. Effects of EYE-ECG training on ECG interpretation skills

Overall, partaking in the study significantly improved participant ECG interpretation skills to moderate effects (pre-post gain BS=4.80±9.10, *t*(90)=5.03, *p*<.03*10^-3^, Cohen’s *d*=1.27; RS=10.45±10.29, *t*(90)=9.68, *p*<.01*10^-13^, *d*=1.19; CS=15.97±9.02, *t*(90)=16.88, *p*<.03*10^-14^, *d*=1.60). Crucially, however, calculation of differential learning gains of INT vs. TAU showed that BS *∆M*=2.14, RS *∆M*=1.25, and CS *∆M*=2.19. This indicated higher, yet statistically non-significant additional improvement in students’ ECG interpretation skills after having seen the EYE-ECG video (BS Welch-*t*(80.17)=1.11, *p*<.27, *d*=0.23; BS Welch-*t*(87.09)=0.58, *p*<.57, *d*=0.12; CS Welch-*t*(80.82)=1.15, *p*<.25, *d*=0.24) (see table 1 (Tab. 1)) .

### 3.3. Identification of best predictors for specific ECG interpretation skill scores

Regression models (for full results see attachment 2 , point D) showed that overall, variables meaningfully accounted for post-test scores (all models highly significant) and contributed a substantial proportion of variance (*R^2^*=.44-.63). Simplification to 5-7 predictors greatly increased parsimony (*R^2^≈R^2^_adj_*) and only led to a relatively small reduction of predictive power in final models as compared to full models. As expected, pre-test scores significantly contributed in all three final models. Of particular interest was the combination of additional significant final predictors: Relevant to BS in this regard was a prior cardiological clerkship, interest in ECGs, accessibility of material, and scores on clinical case 1. Interestingly, having experienced these typically month-long cardiology clerkships only conferred an advantage of 3.23%. In the same vein, judging the provided learning materials as easier was in fact associated with reduced performance, which might be indicative of a more cavalier attitude towards tasks perceived as trivial. Final RS were also predicted by interest in ECGs and case study 1, final CS also by interest in ECGs only. Thus, performing well on the first learning scenario appeared to be of marked importance both for participants’ initial recognition of relevant ECG features (BS) and general strategic weighing of correct against false options (RS) but not for understanding practical ECG scenarios in their entirety (CS) (see table 3 (Tab. 3)).

## 4. Discussion

The present study was based on the proposition that ECG interpretation skills are part of the core skill set for medical students, and that there is both a clear need and the potential to better understand factors that facilitate the successful acquisition of these ECG interpretation skills. Thus, the goal of the present study was twofold:

First, to contribute to the continued improvement of ECG teaching via technology-enhanced presentation of actual expert ECG diagnosis. A novel instructional video visualising expert gaze patterns during ECG interpretation and providing cued retrospective reporting (CRR) audio commentary was developed and its benefit evaluated in this learning intervention study.

Second, to supplement this perspective on the scope of the new instructional design, broader information on participant characteristics, objective and subjective learning parameters, participant involvement, and the impact of individual learning scenarios were gathered. This was evaluated against three different strategies to formally compute successful ECG interpretation skills from the tests administered during the study. Adding the results of these student-based factors may provide some of the background upon which the intervention-based skill gain took place.

Addressing the first research goal, with respect to the video intervention, results showed a small positive point difference of the CRR teaching method against training as usual after only 9 minutes of presentation. That, however, was too small to carry through based on current sample size and effect of prior theoretical ECG knowledge. Given that satisfactory acquisition of ECG interpretation skills is a multi-year process, this finding is very promising and results indicate that being let in on an expert’s real-time inspection pattern and associated thought processes during ECG interpretation may be further developed as a useful learning and teaching tool.

Regarding the potential benefit of using the CRR method for ECG teaching, participant feedback relating to the intervention was overwhelmingly positive and there was general agreement that the verbal content of CRR videos was helpful. Several students however suggested that pausing eye gaze (as opposed to pausing the entire video track) at crucial points instead of showing a continuous loop – i.e., holding the focus spot in place while playing the corresponding auditory explanation – would afford them more time to retrace and comprehend the visual information referred to and is an exciting strategy to be investigated as the next step in a follow-up study.

Concerning the second research aim of elucidating students’ personal background, regression analyses on three conceptually complementary post-learning ECG interpretation skill scores representing the aspects of ECG feature identification, strategic answering, and a full understanding of patient presentations were performed. The presence of some nonsignificant predictors in the final RS and CS regressions – denoting no sufficiently certain contribution despite indicative tendencies for effects – may be interpreted as either due to the relatively small sample size for multivariate linear regression, or as a signal for an association with presently unexplored concepts. Comparison of the three models helped to first illustrate which combination of predictors is generally of importance in predicting students’ ECG interpretation skills (e.g., interest in ECGs). Second, some unique characteristics were identified (e.g., prior cardiological clerkship for prediction of the basic score) that are associated with ECG interpretation skill outcomes only when specific performance aspects (e.g., successful feature detection) are considered. These participant data may therefore provide some leverage in tailoring teaching approaches in a manner that is most enticing to students and may encourage specific outcomes (e.g., full comprehension vs. feature detection) over others.

Similarly, specifically the result of a ubiquitous role of self-proclaimed interest in ECGs in all regression models was unsurprising and highlights both the opportunity and the necessity for instructors to kindle curiosity in the subject, over and above a more limited skill focus as was operationalised by prior knowledge (pre-test scores).

Another specific finding meriting further discussion is that of a significant predictive influence of clinical case 1 for which two alternative underlying explanations – namely order effects or saliency of content – are plausible: Either starting off well into the feedbacked learning component was generally important, or else scenario 1 (posterior myocardial infarction) represented gatekeeper content that fundamentally needed to be understood before further progression of students’ ECG interpretation skills. This unknown could be addressed by randomising presentation of the clinical cases in the future. 

Finally, in thinking about the just discussed points in relation to the first research question, being knowledgeable on sample characteristics may indeed assist the evaluation and further development of the EYE-ECG video for ECG training of medical students: In the present design, participants and the ECG interpretation skills they held initially were randomly sampled in order to best investigate potential changes attributable to the intervention. However, beyond demonstrating such training effect, it is also of great practical relevance to determine which other co-occurring factors may help to optimise the utility of CRR videos. To pick just one of many examples where this could be relevant, it is conceivable that CRR could be more beneficial to absolute beginners (signified by very low prior ECG interpretation skills), who would not be able to spot relevant ECG features from disjointed visual or auditory instruction alone, and hence particularly profit from the concurrent CRR presentation. Inversely, the opposite argument may also be made that it could be particularly the more advanced students who are better equipped to make use of the spontaneous real-time expert video commentary. 

## 5. Limitations

Participant characteristics indicate the study may have had some self-selection tendencies concerning the participant pool. On one end of the spectrum, a relatively high percentage had completed a cardiological clerkship, which may have been due to their greater willingness to volunteer for this thematically related study because cardiology was of great personal interest to those students. On the other end, an equally large proportion reported never even having received any dedicated ECG training. This assertion is demonstrably incorrect (based on recruitment/ inclusion criteria and the university’s syllabus) but is consistent with verbal communication from many participants of feeling a great sense of overload, unpreparedness, dread, and despair towards ECG interpretation in general. Thus, it is of some relevance that this study included both self-reported and performance-based ECG measures.

Further, an interesting question which the current study was not equipped to answer is that of how much ECG interpretation skill is required to derive the greatest benefit from eye-tracking CRR video presentations. While this specific issue cannot be resolved definitively with current data at this point in time, some of the stimulus adjustments proposed above may begin to address this question and could be incorporated in future follow-up studies. Present findings do however extend previous applications of CRR [15], [17], [19], [20], [21] by showing its appropriateness for a medical learning setting.

## 6. Conclusion

One theme that did emerge clearly from the data on students’ personal background is that interest in ECGs plays an important role for the acquisition of ECG interpretation skills, and that propitiously, the EYE-ECG video was perceived as both interesting and useful by participants, which in turn indicates the continued development of CRR videos for the teaching of ECG interpretation will be of value to medical students.

Tangible plans to further expand on the promising initial findings of greater ECG interpretation skill gains in students receiving the CRR video training are: first, to modify and improve the video by incorporating student feedback from this study; second to randomise presentation of clinical cases in order to more fully understand the contribution of specific content on learning outcomes; and third, to further explore to which target group and in which setting CRR videos can provide the greatest benefit.

## Authors’ ORCIDs

- Aline D. Scherff: [0000-0002-7420-2292]
- Stefan Kääb: [0000-0001-8824-3581]
- Martin R. Fischer: [0000-0002-5299-5025]
- Markus Berndt: [0000-0002-4467-5355]

## Competing interests

The authors declare that they have no competing interests. 

## Supplementary Material

EYE-ECG sample video extract (with German audio)

Supplementary material

## Figures and Tables

**Table 1:**
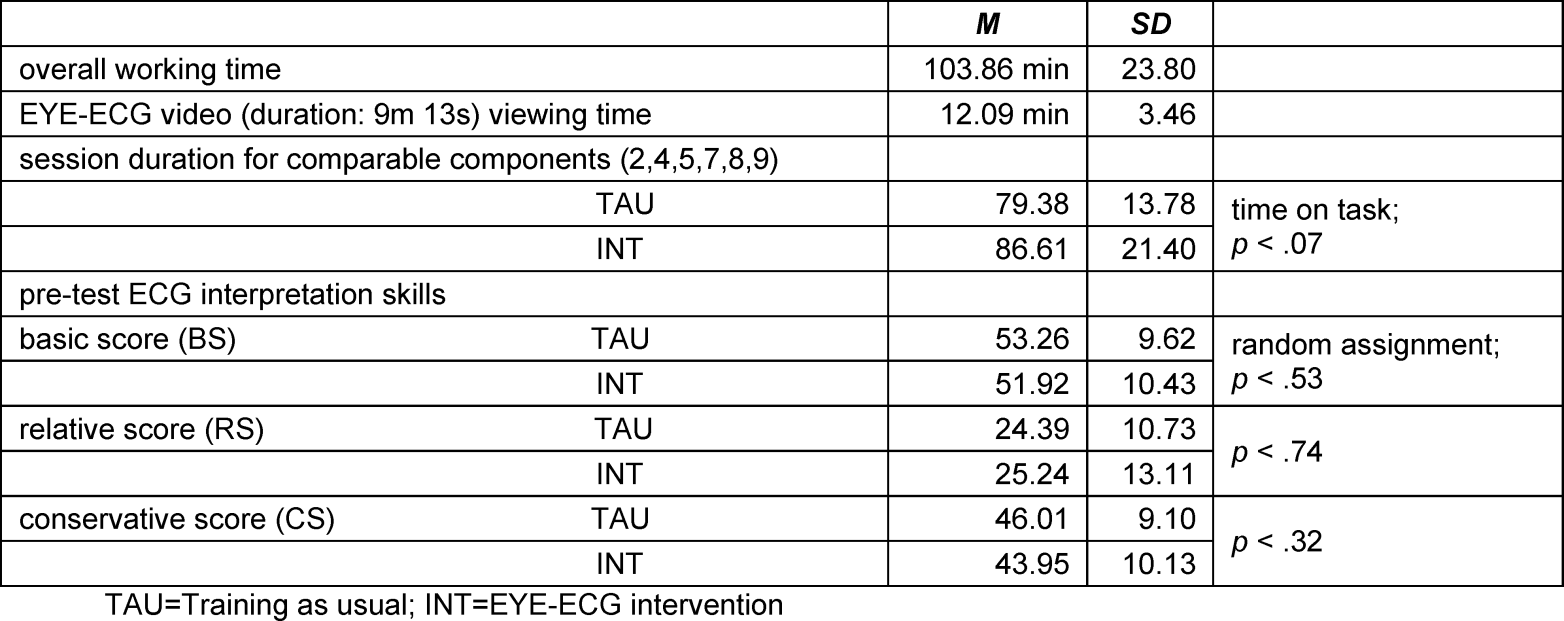
Piloting and manipulation checks

**Table 2:**
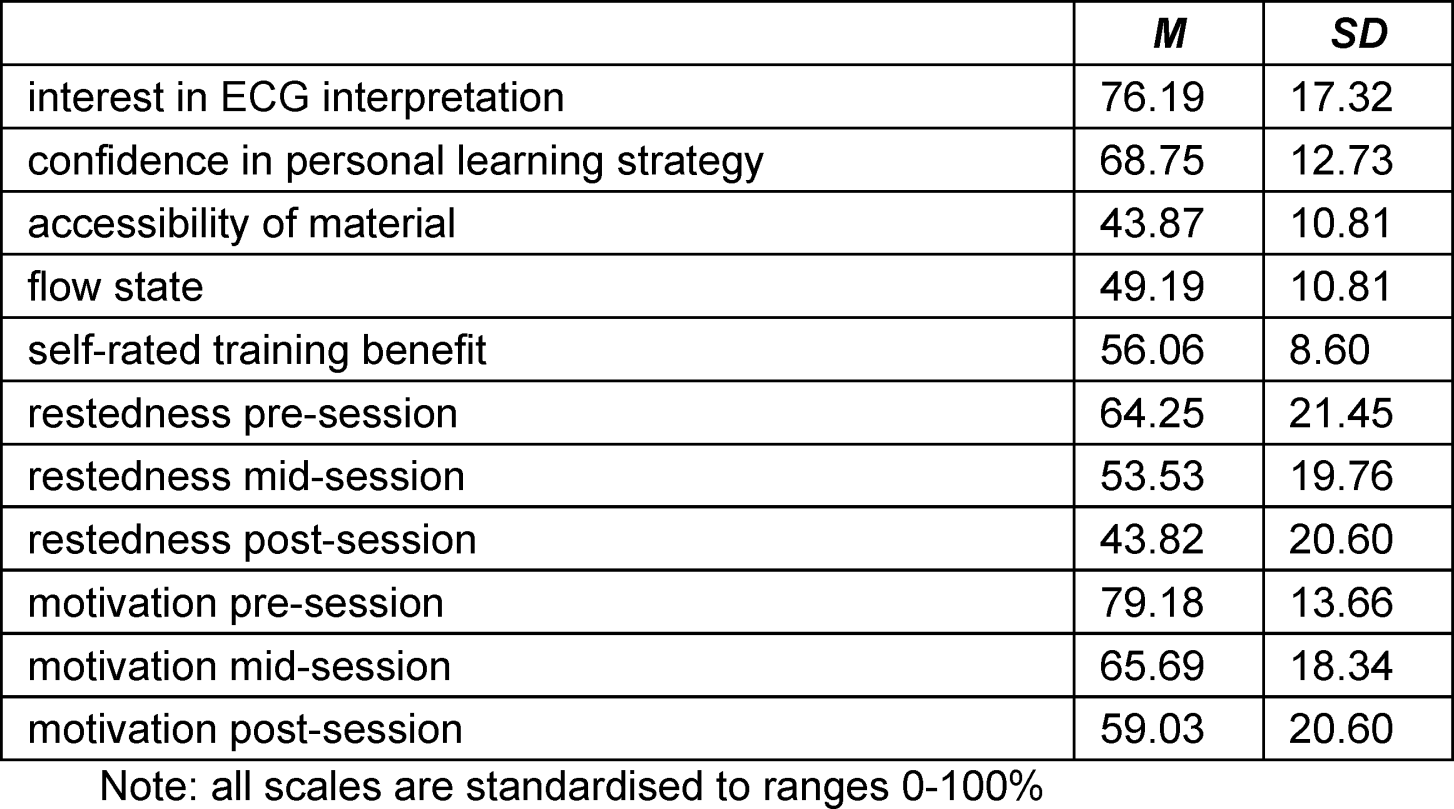
Sample characteristics – additional information

**Table 3:**
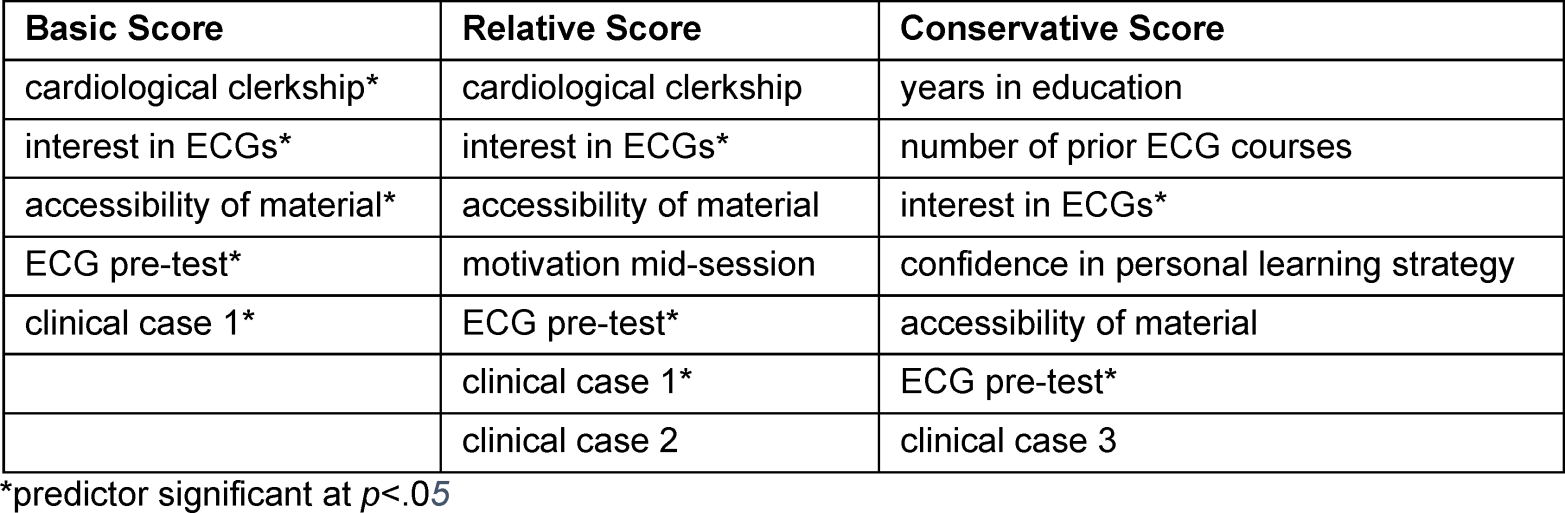
List of predictors included in the 3 final regression models
