# Supplementary material for: EYE-ECG: An RCT of the influence of student characteristics and expert eye-tracking videos with cued retrospective reporting on students’ ECG interpretation skills
Source: GMS J Med Educ. 2024 Sep 16;41(4):Doc40. doi: 10.3205/zma001695 (PMC11474649; doi:10.3205/zma001695)
Supplement: Supplementary material [file JME-41-40-s-002.pdf]

Attachment 2: Supplementary material

Point A

I. Material development phase

Eye-tracking recordings

ECG selection. Diagnostic learning goals were derived from a previous multi-step Delphi study (1) collating expert consensus on expected ECG interpretation skills for medical students in their final clinical year. Accordingly, 15 relevant (anonymized) real-patient ECGs representing typical electrophysiological features and cardiological diagnoses were obtained as high-resolution images. Specifically, these were: normal (healthy) ECG/ ventricular tachycardia/ bradycardia, anterior/ anterolateral/ posterior ST-elevation myocardial infarction, pacemaker ECG with supraventricular/ ventricular stimulation, ECG with supraventricular/ ventricular extrasystoles, complete/ incomplete left bundle branch block, incomplete right bundle branch block, abnormal myocardial repolarization ECG, atrial fibrillation, first-degree atrioventricular block.

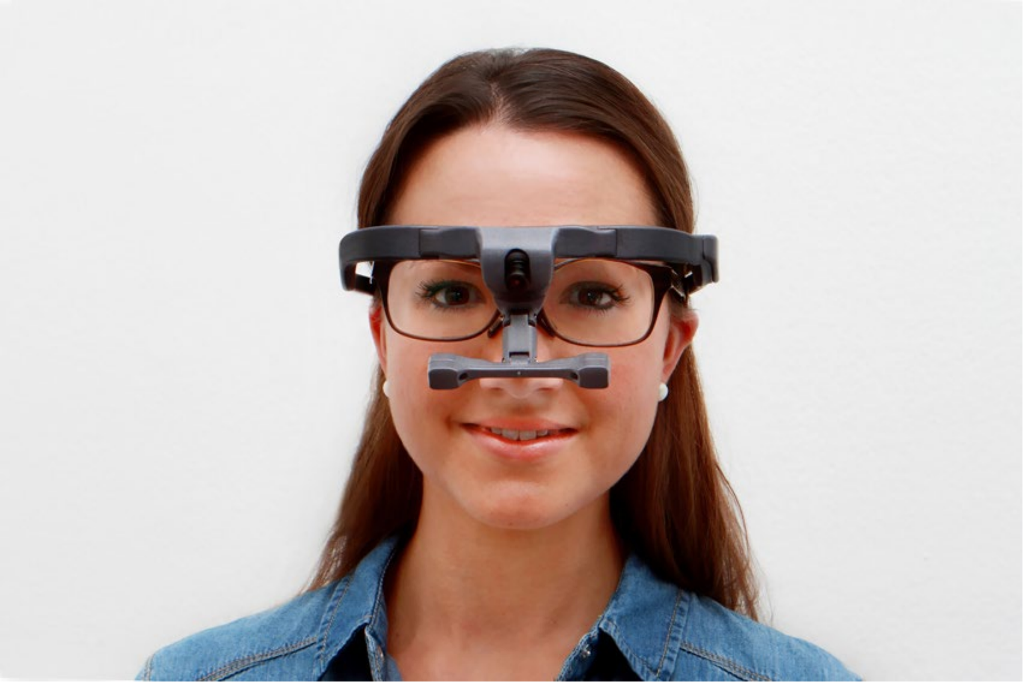

Figure 1 Eye-tracker used in this study (Ergoneers Dikablis Glasses 3, with permission)

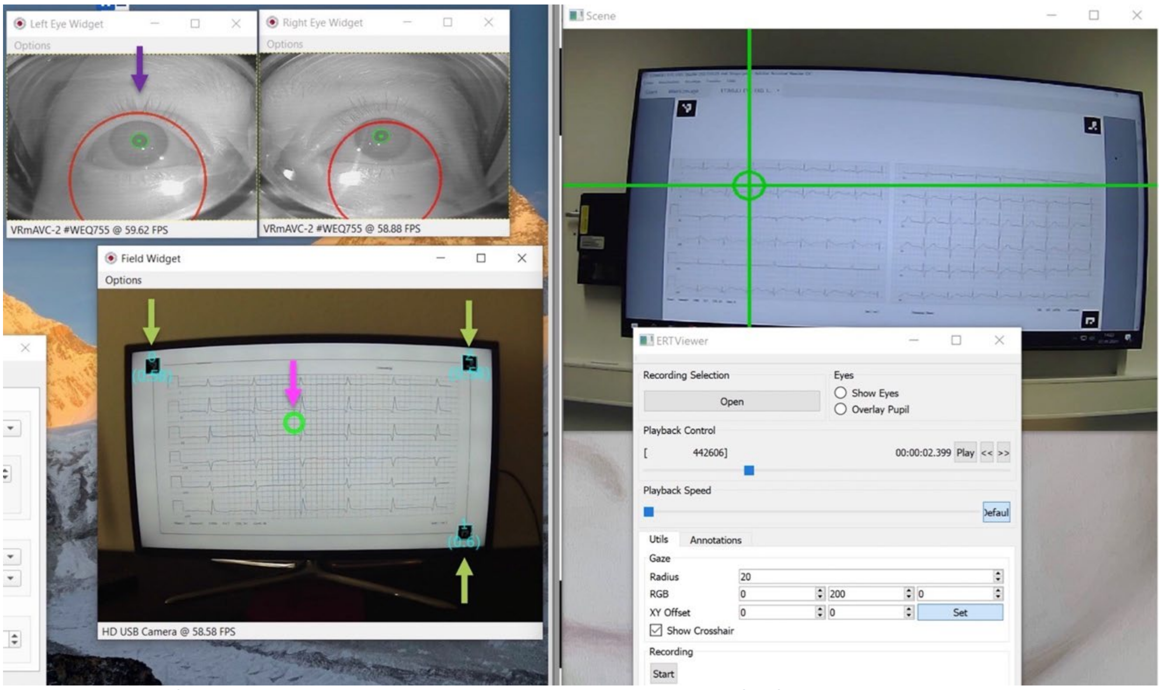

Figure 2 Left side: EyeRecToo. Indicators: purple = detection of left eye; green = ArUco markers; pink = marker showing eye gaze. Right side: ERTViewer.

Eye-tracking. A senior cardiology expert wearing a head-mounted eye-tracker (see Fig. 1) was instructed to silently and freely interpret all ECGs and then verbally state the diagnoses (to signify having reached a conclusion). Quiet interpretation allows for perusal unaffected by divided attention, as one may impair the other (2, 3). Each of the 15 recordings started and finished on an empty screen showing a central fixation cross. ECGs were shown and eye-

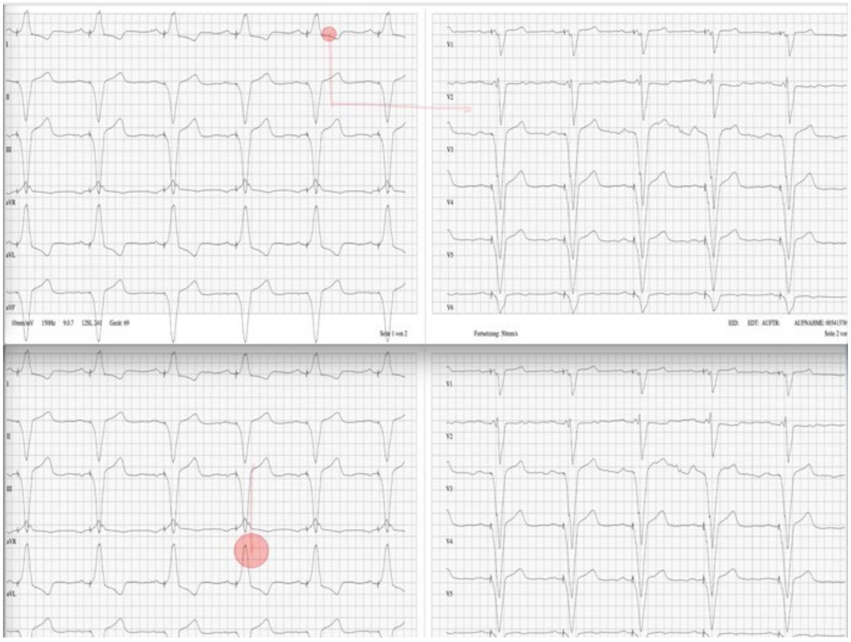

Figure 3 Visualisation of two moments during eye-tracked ECG interpretation

Attachment 1 to Scherff AD, Kääb S, Fischer MR, Berndt M. *EYE-ECG: An RCT of the influence of student characteristics and expert eye-tracking videos with cued retrospective reporting on students' ECG interpretation skills.* GMS J Med Educ. 2024;41(4):Doc40. DOI: 10.3205/zma001695

tracking patterns recorded only during the uninterrupted quiet visual search and interpretation process. Ergoneers Dikablis Glasses 3 hardware and open-source software EyeRecToo (4) were used for recording and data acquisition (see Fig. 2). A custom Python script (using Python 3.8 in Spyder 5.4.3; packages used were numpy, pandas, matplotlib.pyplot, copy, scipy.stats, time) was created for post-processing of raw sensor data (i.e., gaze path smoothing during eye-blinks, adjustment for head position via ArUco (5) markers, extraction of eye and field markers from original recordings and projection onto ECG images directly for subsequent high-quality viewing experience). Quality control was conducted comparing frame-by-frame original recordings on ERTViewer (6) with Python projections and showed identical positions/ paths on visible ECG features; eye-tracking accuracy achieved after calibration was in line with current technical limits at 0.5-1.0° visual angle (7).

Cued retrospective reporting recordings

Video clips. In preparation for CRR audio commentary collection, 15 short video clips were created showing the expert's gaze behavior on each ECG, their length of each 8-27s depending on prior eye-tracking duration. As suggested by Jarodzka and colleagues (8), expert eye gaze was visualized (using Python) as a moving red focus spot with a 500ms trail marking previously viewed locations. Spot size dynamically varied with gaze intensity (dwell time derived from underlying Gaussian heatmap), such that longer focused inspection time was apparent from larger circles. In distinction to Jarodzka et al.'s opaque spots, it was decided to make spots translucent to allow inspected ECG features to be more easily verifiable (see Fig. 3).

Audio commentary. The 15 videos were then presented to the same expert as visual cues accompanied with the request to retrospectively verbally explain (= CRR) their own viewing behavior and the diagnostic processes during the interpretation of the ECGs (e.g., thought processes, strategy, diagnostic information). Individual silent clips were played in real-time (i.e., at original speed) in a constant loop and spontaneous unrehearsed explications of each ECG were recorded until the expert concluded. Briefly, in terms of content generated, the looping videos triggered both verbal explanation of ECG features precisely at the time they were highlighted, and also longer trains of thought on viewing patterns.

Point B

Statistical analyses

All analyses were performed using R (RStudio Cherry Blossom, using readxl, plyr, stringr, car, tidyr, dplyr, reshape2, ggplot2, ggcorrplor, psych packages). Groupwise comparisons of INT vs. TAU were conducted using Welch's t-tests (9); pre-post comparisons were conducted using paired samples t-tests. Basic assumptions were tested and deemed sufficiently met. Model building for student ECG interpretation skills was approached empirically (i.e., bottom-up), exploring which combination of student characteristics best predicted post-test scores. As a baseline, all 26 predictors collected including participant characteristics, objective and subjective learning factors, involvement, clinical cases, pre-test scores, and the learning intervention were entered into a full model with each of the three outcome scores (BS, RS, CS). This permitted an estimation of the extent of variance explained in student ECG interpretation skills when there is rich data on both the students and their answering strategy. A reduced model was then derived by stepwise removal of least predictive contributing factors while also maintaining the largest possible explanatory power until a final solution was reached, as manifested statistically by Akaike information criterion (AIC). These final models allowed some insight into which student attribute or learning component should be primarily addressed to optimise ECG interpretation skills in medical students specifically tailored to the answering strategy desired.

Attachment 1 to Scherff AD, Kääb S, Fischer MR, Berndt M. *EYE-ECG: An RCT of the influence of student characteristics and expert eye-tracking videos with cued retrospective reporting on students' ECG interpretation skills.* GMS J Med Educ. 2024;41(4):Doc40. DOI: 10.3205/zma001695

Point C

Bivariate associations of model variables:

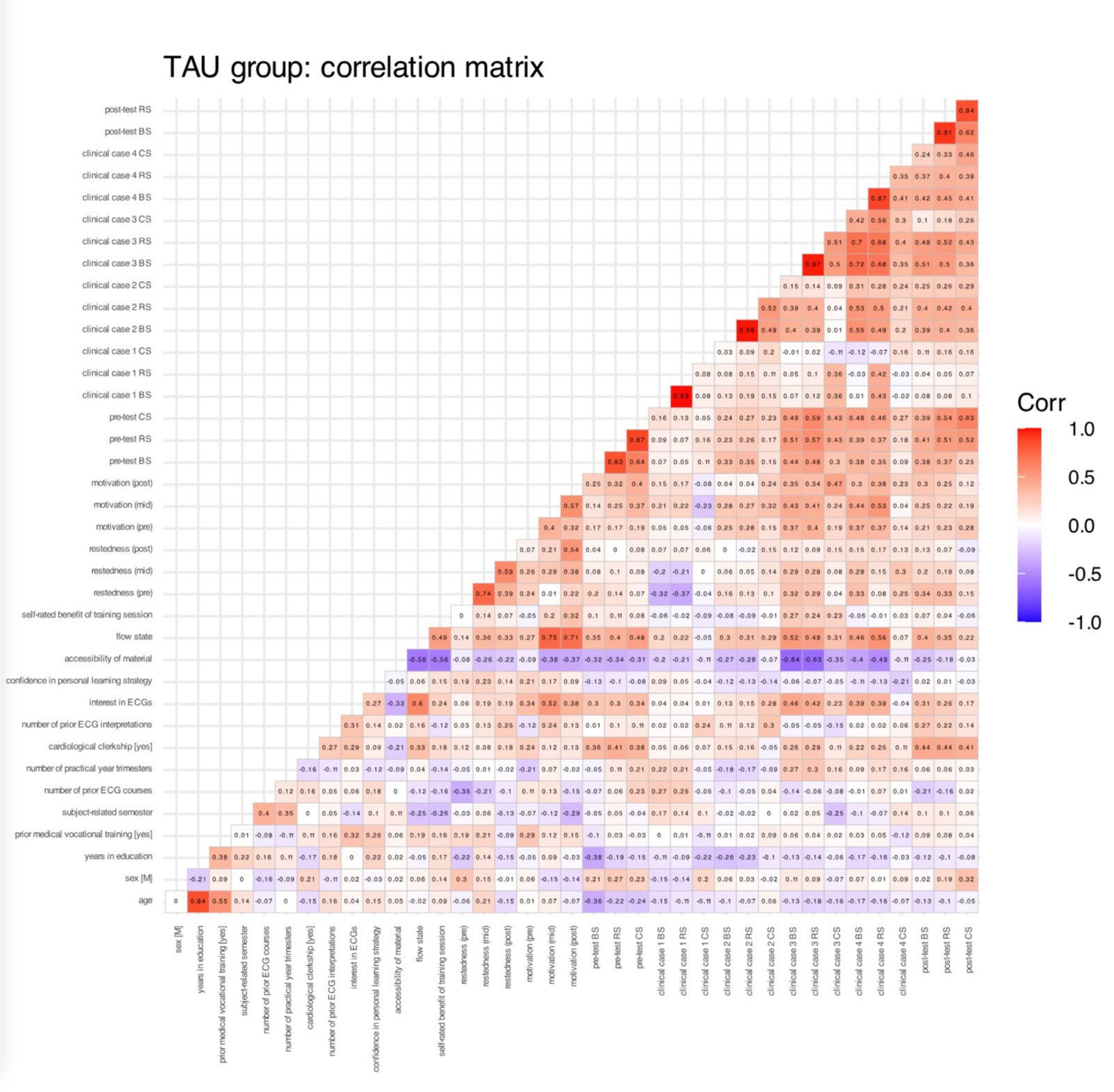

“Treatment as Usual group”: The 38 variables shown and their order corresponds to those used in regression models. Effect sizes are Pearson correlations/ point biserial correlations. [] refers to the reference category. BS = basic score, RS = relative score, CS = conservative score. Freely scalable SVG graphic available from first author.

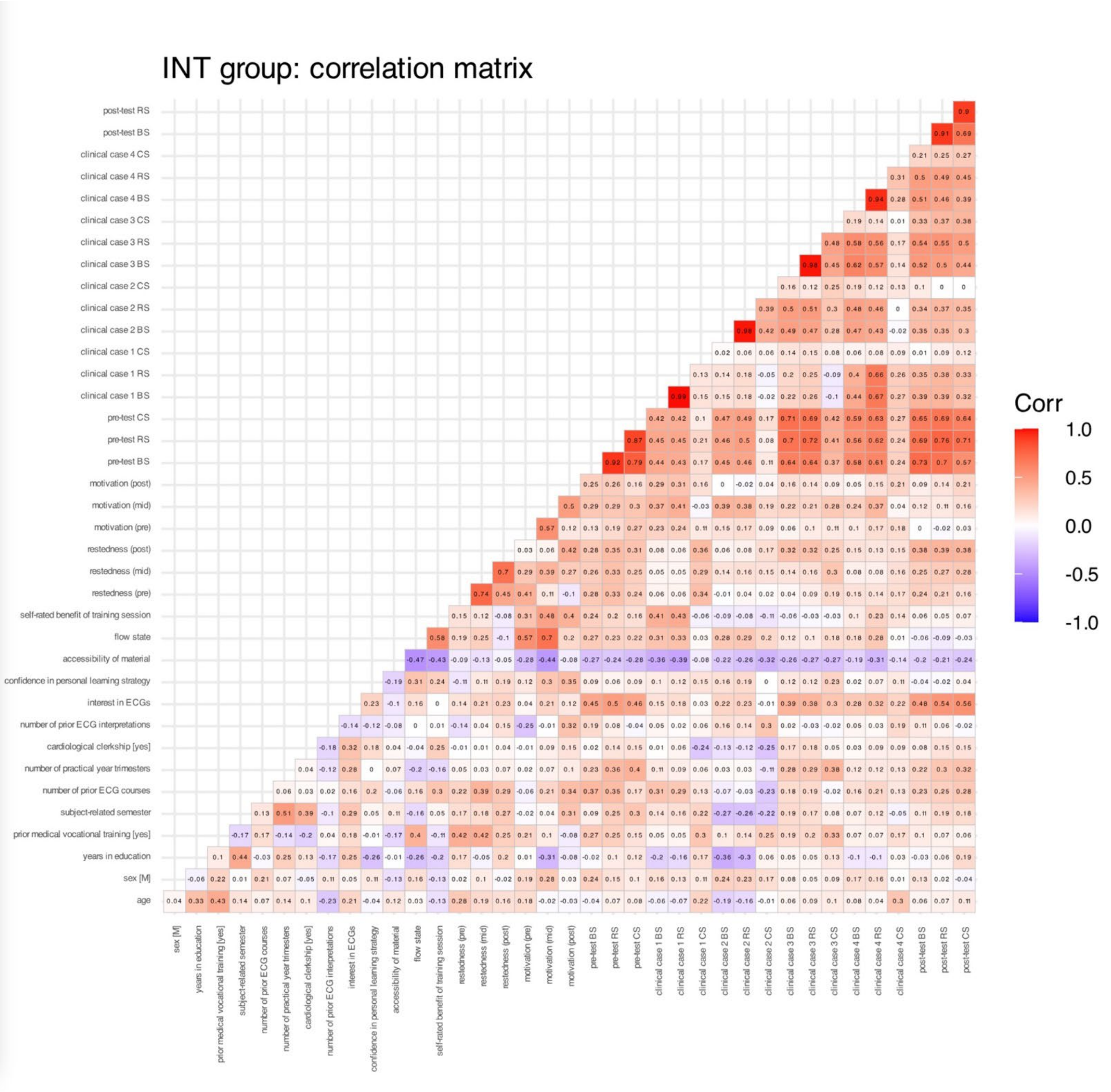

EYE-ECG/ "Intervention group": The 38 variables shown and their order corresponds to those used in regression models. Effect sizes are Pearson correlations/ point biserial correlations. [] refers to the reference category. BS = basic score, RS = relative score, CS = conservative score. Freely scalable SVG graphic available from first author.

Attachment 1 to Scherff AD, Kääb S, Fischer MR, Berndt M. *EYE-ECG: An RCT of the influence of student characteristics and expert eye-tracking videos with cued retrospective reporting on students' ECG interpretation skills.* GMS J Med Educ. 2024;41(4):Doc40. DOI: 10.3205/zma001695

Point D

Table of regression model outcomes for the three ECG interpretation skill scores:

	Specific ECG interpretation skill score	I. Basic Score				II. Relative Score				III. Conservative Score			
		full model		final model		full model		final model		full model		final model	
conceptual grouping	↓ **Included variables (predictors)**	beta	p-value	beta	p-value	beta	p-value	beta	p-value	beta	p-value	beta	p-value
	(intercept)	20.05	.24	**18.27**	**< .01**	0.79	.97	2.91	.62	**51.51**	**$< .04*10^{-1}$**	**50.94**	**$.04*10^{-5}$**
learning intervention	group *[INT]*	2.09	.24			2.30	.27			1.00	.60		
participant characteristics/ obj. characteristics	age	0.12	.77			0.10	.83			-0.20	.66		
	sex *[M]*	-0.83	.68			-0.46	.85			0.08	.97		
	years in education	-0.08	.88			0.16	.80			-0.29	.59	-0.52	.14
	prior medical vocational training *[yes]*	-0.69	.81			-3.30	.34			0.83	.79		
	subject-related semester	-0.24	.75			-0.77	.40			-0.22	.79		
	number of prior ECG courses	-0.84	.55			-0.18	.91			-0.53	.73	0.02	.12
	number of practical year trimesters	-1.50	.53			-0.77	.79			0.84	.75		
	cardiological clerkship *[yes]*	3.09	.11	**3.23**	**< .05**	4.15	.08	3.95	.05	2.08	.32		
	number of prior ECG interpretations	0.01	.45			0.02	.20			0.02	.19		
subj. learning charac-teristics	interest in ECGs	**0.14**	**.03**	**0.14**	**< .01**	**0.20**	**< .01**	**0.20**	**$< .02*10^{-1}$**	**0.16**	**.02**	**0.19**	**$< .06*10^{-2}$**
	confidence in personal learning strategy	-0.07	.29			-0.08	.34			-0.11	.12	-0.09	.16
	accessibility of material	-0.17	.11	**-0.18**	**.02**	-0.17	.17	-0.18	.06	-0.17	.13	-0.13	.10
	flow state	-0.01	.86			< 0.01	.92			-0.03	.74		
	self-rated benefit of training session	0.04	.77			0.04	.76			0.10	.43		
participant involvement	restedness (pre)	0.06	.43			0.06	.47			< 0.01	.92		
	restedness (mid)	-0.03	.73			0.03	.82			-0.02	.83		
	restedness (post)	0.02	.78			-0.03	.73			0.05	.48		
	motivation (pre)	-0.05	.53			-0.05	.59			0.02	.83		
	motivation (mid)	< -0.01	.93			-0.12	.21	-0.08	.17	0.03	.71		
	motivation (post)	0.03	.67			0.08	.30			< -0.01	.95		
learning content	pre-test	0.23	.05	**0.29**	**$.07*10^{-2}$**	0.23	.08	**0.33**	**$.01*10^{-1}$**	**0.30**	**.01**	**0.34**	**$.02*10^{-2}$**
	clinical case 1	**0.35**	**< .01**	**0.34**	**$.04*10^{-3}$**	**0.38**	**$.03*10^{-1}$**	**0.31**	**$< .01*10^{-1}$**	0.04	.76		
	clinical case 2	0.02	.87			0.10	.32	0.12	.12	0.07	.30		
	clinical case 3	0.13	.28			0.14	.29			0.07	.43	0.12	.07
	clinical case 4	-0.05	.66			-0.07	.57			0.04	.67		
	Summary statistics:	$R^2 = .57$, $R^2_{adj} = .39$	$F(26,63) = 3.19$, $p < .01*10^{-2}$	$R^2 = .50$, $R^2_{adj} = .47$	$F(5,84) = 17.03$, $p < .02*10^{-9}$	$R^2 = .63$, $R^2_{adj} = .47$	$F(26,63) = 4.04$, $p < .03*10^{-4}$	$R^2 = .57$, $R^2_{adj} = .53$	$F(7,82) = 15.39$, $p < .01*10^{-14}$	$R^2 = .49$, $R^2_{adj} = .28$	$F(26,63) = 2.35$, $p < .03*10^{-1}$	$R^2 = .44$, $R^2_{adj} = .39$	$F(7,82) = 9.15$, $p < .03*10^{-6}$

Note: [ ] denotes reference group; calculations of scores (cf. statistical analyses section) for pre-test and clinical cases 1-4 corresponded model-wise to the specific (BS, RS, CS) outcomes regressed upon; model selection stepwise backwards *via AIC; significant predictors are bold*

References Attachment

1. Höger A. Ein kompetenzorientierter Lernzielkatalog für die Elektrokardiogramm-Befundung. München: LMU München; 2016.
2. Jou J, Harris RJ. The effect of divided attention on speech production. Bull Psychon Soc. 1992;30(4):301-304. DOI: 10.3758/BF03330471
3. Hutton S, Tegally D. The effects of dividing attention on smooth pursuit eye tracking. Expl Brain Res. 2005;163(3):306-313. DOI: 10.1007/s00221-004-2171-z
4. Santini T, Fuhl W, Geisler D, Kasneci E. EyeRecToo: Open-source Software for Real-time Pervasive Head-mounted Eye Tracking. in: Proceedings of the 12th International Joint Converences on Computer Vision, Imaging and Computer Graphics Theory and Applications (VISIGRAPP 2017). Setúbal, Portugal: SCITEPRESS - Science and Technology Publications; 2017. p.96-101. DOI: 10.5220/0006224700960101
5. Romero-Ramirez FJ, Muñoz-Salinas R, Medina-Carnicer R. Speeded up detection of squared fiducial markers. Image Vision Comp. 2018;76:38-47. DOI: 10.1016/j.imavis.2018.05.004
6. Universität Tübingen. EyeRecToo. Tübingen: Universität Tübingen; 2019. Zugänglich unter/available from: https://www.hci.uni-tuebingen.de/research/Engagement%20&%20Interaction/eyerectoo.html
7. Feit AM, Williams S, Toledo A, Paradiso A, Kulkarni H, Kane S, Morris MR. Toward everyday gaze input: Accuracy and precision of eye tracking and implications for design. in: CHI'17: Proceedings of the 2017 CHI Conference on Human Factors in Computing Systems. 2017. p.1118-1130. DOI: 10.1145/3025453.3025599
8. Jarodzka H, Scheiter K, Gerjets P, Van Gog T. In the eyes of the beholder: How experts and novices interpret dynamic stimuli. Learn Instruct. 2010;20(2):146-154. DOI: 10.1016/j.learninstruc.2009.02.019
9. Rasch D, Kubinger KD, Moder K. The two-sample t test: pre-testing its assumptions does not pay off. Stat Papers. 2011;52(1):219-231. DOI: 10.1007/s00362-009-0224-x
